# Nematode Parasites of the European Pilchard, *Sardina pilchardus* (Walbaum, 1792): A Genuine Human Hazard?

**DOI:** 10.3390/ani12151877

**Published:** 2022-07-22

**Authors:** Màrius V. Fuentes, Elena Madrid, Laia V. Meliá, Francisco Casañ, Sandra Sáez-Durán, María Trelis, Ángela L. Debenedetti

**Affiliations:** Parasites & Health Research Group, Departament de Farmàcia i Tecnologia Farmacèutica i Parasitologia, Facultat de Farmàcia, Universitat de València, Av. Vicent Andrés Estellés s/n, 46100 València, Spain; elena.madrid@uv.es (E.M.); laivamera@gmail.com (L.V.M.); francp19927@gmail.com (F.C.); sandra.saez@uv.es (S.S.-D.); maria.trelis@uv.es (M.T.); aldebenedetti@gmail.com (Á.L.D.)

**Keywords:** European pilchard, *Sardina pilchardus*, Spain, *Hysterothylacium*, *Anisakis*, human anisakidosis risk

## Abstract

**Simple Summary:**

The European pilchard is one of the most frequently consumed fish species in Mediterranean countries, especially in Italy and Spain, and has been reported as the cause of at least eight human anisakidosis cases in Spain since 1991, the parasitic disease caused by the ingestion of fish or cephalopods infested by the larval stage of anisakid nematodes. With the aim to shed light on the potential human parasitosis risk posed by these nematode larvae, we helminthologically analyzed a total of 350 sardines (European pilchard) captured in the Atlantic Ocean (175 sardines) and the Mediterranean Sea (175 specimens), acquired in various Spanish nationwide supermarket chains. The statistical analysis of some helminth parameters revealed a higher presence of nematodes belonging to the genus *Hysterothylacium* (frequency of parasitation of 24.29%; total mean parasite burden of 2.36), usually considered non-parasitic for humans (only three cases reported worldwide), when compared to nematodes of the genus *Anisakis* (5.71%; 0.16). The human anisakidosis risk after the consumption of raw or undercooked sardines and the role of *Hysterothylacium*, the most frequent nematode, is discussed, providing information to consumers. To avoid human infection by anisakid larval nematodes, the established preventive measures are confirmed and new ones are proposed.

**Abstract:**

The European pilchard is one of the most frequently consumed fish species in Mediterranean countries, especially in Italy and Spain, and has been reported as the cause of at least eight human anisakidosis cases in Spain. With the aim to shed light on the potential human parasitosis risk posed by nematode larvae belonging to families Anisakidae or Raphidascarididae, a total of 350 sardines captured in the Atlantic Ocean (175 specimens) and the Mediterranean Sea (175 specimens), acquired in various Spanish nationwide supermarket chains, were helminthologically analyzed. The statistical analysis of some helminth parameters revealed a higher presence of nematodes belonging to the genus *Hysterothylacium* (prevalence 24.29%; mean abundance of 2.36), usually considered non-parasitic for humans (only three cases reported worldwide), when compared to nematodes of the genus *Anisakis* (5.71%; 0.16). The human anisakidosis risk after the consumption of raw or undercooked sardines and the role of *Hysterothylacium*, the most frequent nematode, is discussed, providing information to consumers. To avoid human infection by anisakid larval nematodes, the established preventive measures are confirmed and new ones are proposed, such as the consumption of sardines preferably caught in the Mediterranean and of small-sized specimens available, and the immediate evisceration after fishing.

## 1. Introduction

The sardine, specifically the European pilchard, *Sardina pilchardus* (Walbaum, 1792) (Clupeidae Cuvier, 1817), is a pelagic fish extensively distributed in the Mediterranean Sea (more common in the Adriatic and in the western part), the Black Sea and the North Atlantic Ocean (from the North Sea to Senegal). Its life-span is around ten years, but it is rarely sold when older than three years, ranging in size from 17 to 20 (25) cm [1].

This fatty fish is one of the most widely consumed fish in Mediterranean countries, especially in Italy and Spain. Although it can be frozen or canned, it is largely sold fresh. Moreover, when this fish is consumed fresh, it should be consumed between one or two days after the catch to avoid its deterioration as its flesh is delicate and difficult to preserve, and is therefore usually preserved in vegetable oil, marinated, salted, smoked or dried [2].

Worldwide, catches of sardines have been estimated to be above 4 million tons in 2015, with 5% caught by the European Union, and 18% of which by Spain, being second after Croatia (25%). In Spain, the main origin of this fish for home consumers is Spain’s own national waters, the Mediterranean (70%) and the Atlantic (20%), and consumption in 2016 was estimated to be above 26,000 tons of fresh sardines (0.59 kg/person/year). Although this fish is available throughout the entire year, its organoleptic qualities and its fatty content are best between the months of July and November [1].

Like many other fish sold, this commonly consumed fish is, more often than not, infested by nematode larvae, in its viscera as well as its musculature. These nematodes, commonly known as anisakids, actually belong to two rather different families: the family Anisakidae Skrjabin and Karokhin, 1945, in the case of nematodes mainly belonging to the genera *Anisakis* Dujardin, 1845, and *Pseudoterranova* Mozgovoi, 1951, and the family Raphidascarididae Hartwich, 1954, in the case of the genus *Hysterothylacium* Ward and Magath, 1917. Besides their different taxonomy at the family level, there is also a clear differentiation concerning their potential as human pathogens, with regard to gastrointestinal anisakidosis as well as allergic anisakidosis [3,4,5,6]. However, the non-pathogenicity of *Hysterothylacium* has still not been completely clarified.

A recent meta-analysis carried out concerning the 1967–2017 period revealed an increase in *Anisakis* spp. abundance in several fish species, which may have implications for human health, the health of marine mammals and the profitability of fisheries [7].

In Europe, Spain, Italy and France are the countries where human anisakidosis cases have increased in the past two decades [8]. Moreover, in Spain, about 8000 new cases have been estimated to occur every year [9].

In Spain, since the first human anisakidosis case reported in 1991 [10], at least eight new cases of anisakidosis through the consumption of sardines have been reported, four of them presenting gastrointestinal symptoms, caused by *Anisakis simplex* (Rudolphi, 1809) and *Pseudoterranova decipines* (Krabbe, 1878), after the consumption of marinated sardines [11,12,13,14], and the other four cases presenting hypersensitivity after the consumption of fresh or canned sardines [4].

The majority of studies carried out on the presence of nematode larvae in the viscera and flesh of sardines sold in Spain have revealed a higher prevalence of nematodes of the genus *Hysterothylacium* and the absence or a scarce presence of nematodes of the genus *Anisakis* [5,15,16,17,18,19,20,21,22,23,24,25]. Of the nine species of the genus *Anisakis* identified worldwide, only *A. simplex* s.s. and *Anisakis pegreffii* Campana-Rouget and Biocca, 1955, have been reported as parasites of the European pilchard, with *A. pegreffii* being the only species identified in the Mediterranean Sea, which seems to be the dominant anisakid species in this basin, and *A. simplex* s.s. in the North-East Atlantic, but also *A. pegreffii* off the Iberian coast [24,26,27,28,29,30]. Concerning *Hysterothylacium* larvae, only *Hysterothylacium aduncum* (Rudolphi, 1802) has been reported in the European pilchard originating from the Mediterranean Sea [5,28,31] and the North-East Atlantic coast [5].

With the aim to shed light on the potential human parasitosis risk posed by nematode larvae belonging to families Anisakidae or Raphidascarididae, through the consumption of sardines in Spain, we conducted an analysis of the parasite infection by nematode larvae in *S. pilchardus* sold in five nationwide supermarket chains, which offer fish originating from the Atlantic and Mediterranean coasts of the Iberian Peninsula. The analysis considers various extrinsic (origin, season and days passed after catch) and intrinsic (fish weight and length) factors able to influence parasite infection and parasite burden, thus offering the consumer a tool to facilitate the choice of fish when purchasing with the goal to mitigate the risk of parasitation, as this information is available at the selling point. Moreover, we discuss whether the hazard to humans is genuine or the nematode larvae found in sardines mainly cause the rejection of the fish by consumers only.

## 2. Materials and Methods

### 2.1. Sample

For this analysis, we selected 350 sardine specimens, specifically the European pilchard (*S. pilchardus*), purchased in branches of four nationwide Spanish supermarket chains in València and its metropolitan area. The fish analyzed herein were obtained from 2007 to 2018 as part of a project concerning the potential risk of human anisakidosis through the consumption of fresh fish in Spain, carried out by the Parasites and Health Research Group of the Department of Pharmacy and Pharmaceutical Technology and Parasitology, at the University of València (Spain).

The area of origin, Atlantic, Food and Agriculture Organization of the United Nations (FAO) zone 27–Atlantic Northeast (subareas 27.4–North Sea, 27.8–Bay of Biscay and 27.9–Portuguese Waters) or zone 37 Mediterranean and Black Sea (subarea 37.1–Western Mediterranean, division 37.1.1–Balearic, as well as the date of capture were taken from the fish crates at the point of purchase. The samples were randomly chosen, being fresh and uneviscerated, and once in the laboratory, these were kept in a fridge at 4 °C until dissection. Each specimen was weighed and the total length was measured. Specimens were also grouped according to the season of capture, distinguishing three periods: autumn, winter and spring. The number of specimens analyzed with respect to the origin and the season of capture is shown in Table 1, which also shows the characterization of the specimens according to weight and length.

In total, 151 specimens were analyzed on the same day of capture, while the other 199 were analyzed between the second and the eleventh day after capture.

### 2.2. Fish Inspection

Each specimen was analyzed for the presence of nematode larvae after conventional dissection. The viscera and flesh of each specimen were placed in two different Petri dishes and examined separately. The viscera were dissected under a stereoscopic microscope, and the flesh, after a previous visual inspection, underwent artificial enzymatic digestion [32]. The product resulting from this digestion was also examined under a stereoscopic microscope.

### 2.3. Morphological Identification

All the nematode larvae found were identified according to the morphological characteristics described in the literature [33,34,35,36]. The main characteristics considered for this classification were ventricle and the presence/absence of structures such as intestinal caeca and esophageal appendix, the arrangement and separation of the digestive tract into the esophagus, the position of the excretory pore and the shape of the tail. However, individual larvae were not molecularly identified, as the study is mainly aimed at informing consumers about the nematode larvae presence in order to minimize the potential risk of infection.

### 2.4. Statistical Analysis

The number of infested hosts, range of parasite infection, prevalence and mean abundance were analyzed according to Bush et al. [37] for the total of nematode larvae found, as well as for each of the genera identified. Moreover, these parameters were also calculated for the site of parasitation (viscera and flesh), the season and origin of capture.

The helminth-ecological study was carried out for the total of nematode larvae found, and, in some cases, separately to assess the human risk for the genera *Hysterothylacium* and *Anisakis*. The analysis comprised the comparison of prevalence by the χ^2^ test and abundances by the Mann–Whitney (U) and Kruskal–Wallis (H) tests, and when comparing site of infection abundances, by the Wilcoxon (Z) test. The potential risk of human anisakidosis was assessed analyzing the influence of intrinsic (weight and length) and extrinsic (origin and season) factors on the prevalence and abundance of both genera of nematode larvae and their presence in the flesh, by binary logistic regression (BLR) for prevalence, showing the odds ratio (OR), and the Spearman test (Rho) for abundance. Furthermore, the potential influence of the days after capture (which corresponds to the theoretical day of consumption, 0–11 days) on the presence of larvae in the flesh was also analyzed by BLR, considering parasitized hosts only. The *p*-value threshold used for statistical significance was *p* < 0.05. Statistical analyses were carried out using StatView 5.0 (SAS Institute Inc., Cary, NC, USA) and SPSS 26.0 for Windows (SPSS Inc., Chicago, IL, USA) software packages.

## 3. Results

The helminthological analysis of the 350 sardines showed the presence of two nematode larva morphotypes belonging to the families Raphidascarididae and Anisakidae, identified as *Hysterothylacium* spp. and *Anisakis* type I, respectively. The total prevalence of nematode larvae was 26.29% (24.29% for *Hysterolthylacium* spp. and 5.71% for *Anisakis* type I), with a mean abundance of 2.53 (2.36 for *Hysterolthylacium* spp. and 0.16 for *Anisakis* type I).

### 3.1. Origin of Catch

Atypically, the origin of sardines did not significantly influence the parameters of the total nematode larvae parasite infection (Table 2 and Table 3), although both prevalence and mean abundance were higher in the sample caught in the Atlantic Ocean than in the Mediterranean Sea. However, although parasite infection by *Anisakis* type I larvae presented low values, significant statistical differences were found between prevalence (χ^2^ = 11.412, *p* = 0.001, df = 1) and mean abundance (U = 14086, *p* = 0.001, df = 1), always being higher in the Atlantic sample with respect to the Mediterranean (OR = 6.17).

### 3.2. Season of Catch

The season of catch (Table 4 and Table 5) significantly influenced nematode larvae infection with regard to both prevalence (χ^2^ = 40.305, *p* < 0.0001, df = 2) and mean abundance (H = 35.396, *p* < 0.0001, df = 2), with winter being the season with the highest prevalence (OR = 9.48 of winter when compared to autumn, OR = 4.58 of spring when compared to autumn, and OR = 2.07 of winter when compared to spring), and spring being the season with the highest worm burden. However, once again, *Anisakis* type I larvae were the exception, without significant differences of parasite infection between the sardines caught in winter and spring.

### 3.3. Size of Sardines

The size of sardines analyzed (weight and length) had a positive correlation with parasite infection, with both prevalence (χ^2^ = 11.022, *p* = 0.001, df = 1, in the case of weight; χ^2^ = 13.781, *p* < 0.0001, df = 1, in the case of length) and abundance (Rho = 0.162, *p* = 0.002, in the case of weight; Rho = 0.210, *p* = 0.0001, in the case of length) of larvae increasing together with the size of the fish.

### 3.4. Site of Infection of Nematode Larvae

The parasite infection (prevalence and abundance) was higher in the viscera when compared to the flesh. Considering the total of nematode larvae, significant statistical differences were found: χ^2^ = 35.305, *p* < 0.0001, df = 1, in the case of prevalence; Z = 6.682, *p* = 0.0001, df = 2 in the case of mean abundance. These results followed the same pattern as the origin and the season of catch.

In the case of *Hysterothylacium* spp., 22 sardines (6.29%) presented nematode larvae in the flesh with a range of 1–12 larvae, and 16 of them also carried larvae in the viscera. The prevalence was highest in sardines caught in the Atlantic and during the winter, but without significant statistical differences. The minimum weight and length of sardines with *Hysterothylacium* spp. larvae in the flesh were 16.20 g and 13.60 cm, respectively. Although parasitation (prevalence and abundance) was higher in the fish caught in the Atlantic Ocean, there were no statistical differences between both origins.

In the case of *Anisakis* type I, only six sardines (1.71%) presented nematode larvae in the flesh, all of them with only one larva, and only two also carrying larvae in the viscera. Three of these sardines were caught in the Atlantic Ocean and the other three in the Mediterranean Sea. Two sardines were caught in autumn, one in winter and three in spring. The minimum weight and length of sardines with *Anisakis* type I larvae in the flesh were 16.20 g and 13.40 cm, respectively.

The number of days that passed between the catch and the analysis (hypothetical consumption) of sardines did not have any effect on the migration of nematode larvae from the viscera to the flesh. No statistical relationship was found.

## 4. Discussion

The parasite infection by nematode larvae observed in the 350 sardines analyzed, purchased at nationwide Spanish supermarket chains, agrees with the great variability previously reported in other studies, both in sardines from the Atlantic Northeast as well as from the Mediterranean. Our results confirm the considerable differences, in general within the same fish species, mainly found with regard to prevalence and seasonality [38,39]. In the present case of sardines, the variations related to the season of capture may be related to the presence of invertebrate intermediate hosts and their definitive hosts (cetaceans in the case of *Anisakis* and cold-blooded marine organisms in the case of *Hysterothylacium* [5].

The studies carried out since the 1990s have reported largely variable prevalence in both *Hysterothylacium* spp. and *Anisakis* spp. in sardines originating from the Atlantic Northeast and the Mediterranean: from 3.4% [5] to 40.0% [18] in the Atlantic Northeast and from 9.1% [5] to 42.4% [29] in the Mediterranean, in the case of *Hysterothylacium* spp.; without parasite infection [5] or from 10% [18] to 28.3 [24], 50% [40] and >60% [41,42] in the Atlantic Northeast, and without parasite infection [24,43] or from 0.2% [28] and 3.3–4.5% [26,44,45] to 13.1% [27], and even to 44.9% [29] and 50% [41] in the Mediterranean in the case of *Anisakis* spp.; and in other studies, even with a total absence of nematode larvae in the Atlantic Northeast [21] as well as in the Mediterranean [22].

The sardine mainly feeds on zooplankton, which includes the invertebrate intermediate hosts of *Anisakis* and *Hysterothylacium* such as copepods and euphausiids, among others. However, the higher parasite infection of sardines with *Hysterothylacium* spp. in comparison to *Anisakis* spp. could suggest that the ideal invertebrate hosts of *Anisakis* spp., euphausiids, are not included in the habitual diet of the sardine or that euphausiids living in the upper layers of the water column, where sardines live and feed, are less parasitized by *Anisakis* spp. larvae [22,46]. Moreover, the results of the present study confirm the higher prevalence of larvae of the genus *Anisakis* in sardines caught in the Atlantic Northeast with respect to those of Mediterranean origin. Although the presence of dolphins and other cetacean definitive hosts of *Anisakis* spp. is higher in Atlantic Northeast waters than in the Mediterranean, which could explain this difference, the current results could also reinforce the hypothesis that the sardine is a susceptible fish to *A. simplex* s.s. larvae, the predominant *Anisakis* species in the Atlantic Northeast, but not quite optimal for *A. pegreffii* larvae, the predominant species in the Mediterranean [4,47].

The higher parasite infection rate of nematode larvae in viscera than in the flesh is a consequence of the fact that viscera are the usual localization in the fish of Anisakidae and Raphidascarididae larvae in fish. However, the prevalence of *Hysterothylacium* spp. larvae (6.29%) and the low prevalence of *Anisakis* type I (1.71%) in the flesh of sardines caught in both origins stand out. Moreover, the presence of larvae of both genera in the flesh was observed even in sardines of small size. This fact can be related to the suggestion that larvae migration to the flesh is facilitated in small-sized fish due to the shorter distance between those two sites of infection, and consequently the shorter distance the larvae have to cover [48].

Although Smith [49] postulated that seasonality does not have a great influence on fish infection by *Anisakis* larvae, the changing feeding habits throughout the year of some fish species, such as anchovies [50], the mackerel [22] and in the present case, the sardine [44], could explain the outstanding seasonal variations in parasitation by *Anisakis* spp. and/or *Hysterothylacium* spp. observed in these fish species.

The influence of the fish size (length and weight) has previously been reported in the sardine [5,24,29,41] as well as in other fish species, showing that the youngest sardines, less parasitized, preferably feed on phytoplankton [46], and the older ones, more parasitized, have changed their feeding habits and also accumulate parasites throughout their lives. On the other hand, the generally lower rate of migration of larvae to the flesh will explain the lack of correlation between this parameter with the number of days passed since the capture of the fish until their analysis (potential consumption), contrary to what has been demonstrated in other fish species such as the blue whiting [25,51], the mackerel [25,52] and the European hake [25,53]. Another explanation could be that, as suggested in other previous studies in the European pilchard [29] and anchovy [54], larvae migrate to the flesh even intra vitam, and consequently, the days passed before consumption may not be as decisive in larval migration in the case of sardines.

### Human Anisakidosis Risk through Sardine Consumption

Human anisakidosis is defined as the gastrointestinal and/or allergic parasitosis caused by nematode larvae belonging to the family Anisakidae. However, although the genus *Hysterothylacium* belongs to the family Raphidascarididae, the few cases of human parasitosis reported worldwide caused by *Hysterothylaicum* larvae, mainly *H. aduncum*, could also be included as human “anisakidosis *sensu lato*” to facilitate the assessment of and the discussion about the human risk through fish consumption. It is also important to consider that *Anisakis*, *Pseudoterranova* and *Contracaecum* (Railliet and Henry, 1912), the recognized anisakid species able to cause “anisakidosis *sensu stricto*” in humans, have been found together with *Hysterothylacium* spp. sharing some host fish species.

In general, the consumption of sardines, even when consumed raw or insufficiently cooked without having been frozen adequately previously, is not considered a human hazard due to the generally low parasite infection parameters of anisakids, mainly *Anisakis* spp., reported in this species in some previous studies. This fact is more relevant in sardines originating from some fishing grounds of the Mediterranean Sea [29,41]. The results of the current study, in which the prevalence of *Anisakis* type I larvae in the flesh is only 1.71% and the mean abundance is 0.02, are also able to support this assumption. Consequently, the human anisakidosis risk through the consumption of sardines should be considered very low, with very few cases of gastro-allergic anisakidosis, mainly anisakiosis, with only eight cases reported in Spain in the past three decades. The proposed human anisakiosis risk through European pilchard consumption is in accordance with other results previously reported [42], classifying the European pilchard caught in the ICES IX (Portuguese coast), as FPR Very Good, indicating very low risk of human anisakiosis. Moreover, the nematode larvae of *Hysterothylacium* genus, in spite of the high prevalence usually found, is considered non-parasitic for humans and not a cause of gastro-allergic parasitosis. However, some insights and facts about these issues should be reconsidered; although focused on Spain, they could be extrapolated to other European countries, such as Italy, France and Portugal, where sardine consumption and the related culinary habits (an uncooked common ingredient of some traditional fish dishes) are relatively similar:

- Since 1991, at least eight cases of anisakidosis (gastrointestinal and/or allergic) have been reported, related to the consumption of fresh, marinated or canned sardines [4,11,12,13,14];
- Numerous reports on nematode larvae in sardines sold in Spain have revealed the scarce presence or even the absence of *Anisakis* spp. and a high prevalence of *Hysterothylacium* spp. larvae [5,15,16,17,18,19,20,21,22,23,24,25];
- There have been at least three human cases of hysterothylaciosis reported in humans, the last one being an invasive gastric case reported few years ago in Spain, after the consumption of raw fish [55,56,57];
- Some authors have demonstrated that *H. aduncum* has antigens in common with *A. simplex*, which could be implicated in similar allergic processes [3,6,58], although human cases of allergic parasitosis, due to *Hysterothylacium* spp., have not been reported;
- The current analysis, similar to previous studies, reports a total prevalence of 7.43% and a mean abundance of 0.20 (range 1–12) of nematode larvae in the flesh, the most frequently consumed part of sardines.

Consequently, the consumption of sardines should not be underestimated as an actual hazard of human “anisakidosis *sensu lato*”, including hysterothylaciosis, after considering: the large number of human anisakidosis cases estimated to occur annually in Spain and other European countries; the recognition of the sardine as a source of anisakidosis; the potential of *Hysterothylacium* spp. larvae to cause gastro-allergic human hysterothylaciosis; the possibility of *Hysterothylacium* spp. larvae to migrate to the flesh (the most frequently consumed part of the sardine); and the fact that visual fish inspection, usually at random, carried out by food business operators at fish markets or other selling points is not sensitive enough and, therefore, does not guarantee the absence of larvae in the flesh, particularly in the case of *Hysterothylacium* spp., due to its smaller size compared to *Anisakis* spp., with which it can be easily confused, especially by non-experts such as consumers.

## 5. Conclusions

Nematode parasite larvae of the sardine should be considered a potential hazard of human gastro-allergic “anisakidosis *sensu lato*”, and consequently, all preventive measures established for other fish species should be applied by consumers, following European regulations concerning preventive measures to inactivate nematode larvae [59]: freezing fish at −20 °C at least for 24 h or at −35 °C for at least 15 h in the case of fish meant to be consumed raw or undercooked, and cooking the piece until a core temperature of 60–70 °C for 5 to 10 min has been reached. Moreover, consumers are able to minimize the risk of infection through the consumption of sardines preferably caught in the Mediterranean, and through the consumption of the small-sized specimens available. It is strongly recommended that consumers consult the product label, including traceability data, as regulated by the European Commission [60,61], which should be provided by the supermarket upon consumer request. Furthermore, although the number of days passed since the capture of the fish until their analysis (potential consumption) did not influence the parasite infection of the flesh, and sardines are usually consumed rapidly, often within one day due to its short shelf life, the immediate evisceration after fishing is recommended to minimize the potential post mortem migration of larvae from the viscera to the flesh.

It is essential to carry out more studies concerning the pathogenicity of *Hysterothylacium* spp. larvae in humans and the tracing of nematode larvae infection at the species level (morphological and molecular identification) in sardines from different FAO zones to be aware of an increase in *Anisakis* spp. or *Hysterothylacium* spp. infection, and the implementation of molecular identification of nematode larvae causing human “anisakidosis *sensu lato*” cases to confirm the species involved.

## Figures and Tables

**Table 1:** Number (N) of sardines analyzed and their characterization by weight (g) and length (cm)—range, mean and standard deviation (SD)—by origin and season of catch.

		Weight			Length		
Origin	N	Range	Mean	SD	Range	Mean	SD
Atlantic	175	8.8–120.2	46.2	24.7	12.7–23.7	17.8	2.9
Mediterranean	175	8.8–120.2	35.9	20.0	12.0–23.5	16.3	2.6
**Season**							
Autumn	102	8.8–81.8	36.6	18.2	12.0–21.5	16.3	2.4
Winter	141	13.7–120.2	41.9	24.1	12.7–23.5	17.4	2.6
Spring	107	8.8–99.2	44.2	25.1	12.0–23.7	17.4	3.4

**Table 2:** Number (N) and prevalence (%) of sardines parasitized by nematode larvae according to the origin of catch.

Nematode Larvae	Origin			
	Atlantic		Mediterranean	
	N	%	N	%
Total nematode	52	29.71	40	22.86
viscera	47	26.86	36	20.57
flesh	14	8.00	12	6.86
*Hysterothylacium* spp.	48	27.43	37	21.14
viscera	44	25.14	35	20.00
flesh	13	7.43	9	5.14
*Anisakis* type I	17	9.71	3	1.71
viscera	15	8.57	1	0.57
flesh	3	1.71	3	1.71

**Table 3:** Mean abundance (mA), mean intensity (mI), standard deviation (SD) and range of nematode larvae in sardines according to their origin of catch.

Nematode Larvae	Origin			
	Atlantic (*n* = 175)		Mediterranean (*n* = 175)	
	mA ± SD (Range)	mI ± SD (Range)	mA ± SD (Range)	mI ± SD (Range)
Total nematode	3.89 ± 17.76 (0–212)	13.10 ± 30.88 (1–212)	1.16 ± 3.87 (0–32)	5.08 ± 6.82 (1–32)
viscera	3.61 ± 17.63 (0–212)	13.43 ± 32.26 (1–212)	1.05 ± 3.76 (0–32)	5.11 ± 7.01 (1–32)
flesh	0.29 ± 1.32 (0–12)	3.57 ± 3.28 (1–12)	0.11 ± 0.54 (0–6)	1.58 ± 1.44 (1–6)
*Hysterothylacium* spp.	3.61 ± 17.38 (0–211)	13.15 ± 31.48 (1–211)	1.12 ± 3.86 (0–32)	5.30 ± 7.03 (1–32)
viscera	3.34 ± 17.26 (0–211)	13.27 ± 32.73 (1–211)	1.03 ± 3.76 (0–32)	5.14 ± 7.41 (1–32)
flesh	0.27 ± 1.31 (0–12)	3.62 ± 3.43 (1–12)	0.09 ± 0.53 (0–6)	1.78 ± 1.64 (1–6)
*Anisakis* type I	0.29 ± 1.44 (0–16)	2.94 ± 3.77 (1–16)	0.04 ± 0.39 (0–5)	2.33 ± 2.31 (1–5)
viscera	0.27 ± 1.43 (0–16)	3.13 ± 3.96 (1–16)	0.02 ± 0.30 (0–4)	4.00 (4)
flesh	0.02 ± 0.13 (0–1)	1.00 (1)	0.02 ± 0.13 (0–1)	1.00 (1)

**Table 4:** Number (N) and prevalence (%) of sardines parasitized by nematode larvae according to the season of catch.

	Seasons					
	Autumn		Winter		Spring	
Nematode Larvae	N	%	N	%	N	%
Total nematode	7	6.86	58	41.13	27	25.23
viscera	6	5.88	52	36.88	25	23.36
flesh	2	1.96	17	12.06	7	6.54
*Hysterothylacium* spp.	4	3.92	56	39.72	25	23.36
viscera	4	3.92	51	36.17	24	22.43
flesh	-	-	16	11.35	6	5.61
*Anisakis* type I	2	1.96	10	7.09	7	6.54
viscera	2	1.96	9	6.38	5	4.67
flesh	2	1.96	1	0.71	3	2.80

**Table 5:** Mean abundance (mA), mean intensity (mI), standard deviation (SD) and range of nematode larvae in sardines according to the season of catch.

	Seasons					
	Autumn (*n* = 102)		Winter (*n* = 141)		Spring (*n* = 197)	
Nematode Larvae	mA ± SD (Range)	mI ± SD (Range)	mA ± SD (Range)	mI ± SD (Range)	mA ± SD (Range)	mI ± SD (Range)
Total nematode	0.15 ± 0.65 (0–5)	2.14 ± 1.46 (1–5)	2.96 ± 7.96 (0–58)	7.21 ± 11.15 (1–58)	4.21 ± 21.36 (0–212)	16.70 ± 40.53 (1–212)
viscera	0.13 ± 0.56 (0–4)	2.17 ± 1.17 (1–4)	2.62 ± 7.78 (0–58)	7.10 ± 11.57 (1–58)	4.05 ± 21.23 (0–212)	17.32 ± 41.85 (1–212)
flesh	0.02 ± 0.14 (0–1)	1.00 (1)	0.35 ± 1.40 (0–12)	2.88 ± 3.08 (1–12)	0.17 ± 0.83 (0–7)	2.57 ± 2.23 (1–7)
*Hysterothylacium* spp.	0.08 ± 0.42 (0–3)	2.00 ± 0.82 (1–3)	2.74 ± 7.18 (0–44)	6.91 ± 10.09 (1–44)	4.04 ± 21.20 (0–211)	17.28 ± 41.79 (1–211)
viscera	0.08 ± 0.42 (0–3)	2.00 ± 0.82 (1–3)	2.40 ± 7.00 (0–44)	6.65 ± 10.41 (1–44)	3.90 ± 21.09 (0–211)	17.38 ± 42.49 (1–211)
flesh	-	-	0.34 ± 1.40 (0–12)	3.00 ± 3.14 (1–12)	0.14 ± 0.79 (0–7)	2.50 ± 2.51 (1–7)
*Anisakis* type I	0.07 ± 0.51 (0–5)	2.33 ± 2.31 (1–5)	0.22 ± 1.42 (0–16)	3.10 ± 4.61 (1–16)	0.18 ± 0.89 (0–7)	2.71 ± 2.43 (1–7)
viscera	0.05 ± 0.41 (0–4)	2.50 ± 2.12 (1–4)	0.21 ± 1.41 (0–16)	3.33 ± 4.82 (1–16)	0.15 ± 0.86 (0–7)	3.20 ± 2.68 (1–7)
flesh	0.02 ± 0.14 (0–1)	1.00 (1)	0.01 ± 0.08 (0–1)	1.00 (1)	0.03 ± 0.17 (0–1)	1.00 (1)

## Data Availability

The database used to carry out the present study is not publicly available due to internal policy of our departments. However, the database could be available, after a justification of its use, upon request to the corresponding author.
